# Ultrasound-guided anterior iliopsoas muscle space block effectively reduces intraoperative hypotension in elderly adults undergoing hip surgery: A randomised controlled trial

**DOI:** 10.3389/fnmol.2023.1119667

**Published:** 2023-01-23

**Authors:** Qingyu Teng, Chengyu Wang, Jing Dong, Hai Yan, Moxi Chen, Tao Xu

**Affiliations:** ^1^Department of Anaesthesiology, Shanghai Sixth People’s Hospital Affiliated to Shanghai Jiao Tong University School of Medicine, Shanghai, China; ^2^Department of Anaesthesiology, Suzhou Hospital of Anhui Medical University, Suzhou, Anhui, China

**Keywords:** hip fracture surgery, iliopsoas space block, intraoperative hypotension, perioperative analgesia, lumbosacral plexus block

## Abstract

**Background:**

Hypotension often occurs during hip surgery in elderly adults with conventional posterior lumbosacral plexus block.

**Purpose:**

We conducted a randomised controlled trial to determine if simple iliopsoas space block can lower the incidence of intraoperative hypotension (IOH) and provide sufficient perioperative pain relief during hip fracture surgery in elderly adults.

**Methods:**

Patients undergoing surgery for elderly hip fracture were randomised to receive either an anterior iliopsoas space block with a lateral femoral cutaneous nerve block or a posterior lumbosacral plexus block. The primary outcome was a composite measure of IOH incidence comprising frequency, absolute and relative hypotension durations.

**Results:**

Compared to the posterior group, the iliopsoas space block group had a decreased median frequency of IOH [1.09 (0–2. 14) vs. 3 (1.6–4.8), *p* = 0.001, respectively] along with lower absolute [5 (0–10) min] and relative [minutes below systolic blood pressure of 100 mmHg in % of total anaesthesia time, 6.67 (0–7.65)] duration of IOH compared to the posterior group [35 (10–45) min, *p* = 0.008; 37.6 (12.99–66.18), *p* = 0.004, respectively]. The median pain levels in the post-anaesthesia care unit and median intraoperative sufentanil usage were comparable between the iliopsoas space group [2 (1–3); 8 (6–10) μg] and posterior group [1 (0–3); 5 (5–8) μg]. Thermal imaging revealed that the limb injected with the iliopsoas space block had a higher skin temperature than the unblocked limb in the sacral plexus innervated region.

**Conclusion:**

A single iliopsoas space block lowers the IOH incidence and provides comparable perioperative analgesia to conventional lumbosacral plexus block.

**Clinical Trial Registration:**

Trial registration at www.chictr.org.cn (ChiCTR2100051394); registered 22 September 2021.

## Introduction

One of the most common fractures in senior people are hip fractures, which affect millions of people every year, causing pain, disability, and socioeconomic difficulty (Tajeu et al., 2014). The best therapy for hip fracture, particularly in the elderly, is internal fixation and arthroplasty, which permits early ambulation and decreases the likelihood of fracture consequences (Bhandari and Swiontkowski, 2017).

For hip fracture surgery, in addition to endotracheal-intubation, general and spinal anaesthesia, the lumbosacral plexus combined with laryngeal-mask general anaesthesia is currently widely promoted, especially for high-risk patients (de Visme et al., 2000; Ho and Karmakar, 2002; Strid et al., 2017). Compared with the previous two techniques, conventional posterior lumbosacral plexus block with laryngeal-mask general anaesthesia provided adequate pain relief during surgery, shortened hospital stays, and decreased mortality (Chen et al., 2018; Scurrah et al., 2018). However, the posterior lumbosacral plexus also has some disadvantages, such as multiple puncture injections, turning over to change the patient’s position, and intraoperative hypotension (IOH; Aksoy et al., 2014; Zhang et al., 2022), which may be due to the diffusing local anaesthetics into the epidural area, sympathetic nerve block, or excessive analgesia (Lee and Braehler, 2017).

IOH is often used to characterise arterial hypotension in patients undergoing surgery under general anaesthesia (Bijker et al., 2007). In patients undergoing general surgery, neurological surgery, geriatric hip surgery, or cardiovascular surgery, reductions in arterial blood pressure below the lower limit of the vascular autoregulation curve are related to ischemia of vital organs (Bayliss, 1902; Mosher et al., 1964; Harper, 1966) and adverse organ outcomes (e.g., myocardial injury, stroke, and acute renal injury; Bijker et al., 2012; Aronson et al., 2013; Walsh et al., 2013). In addition, IOH is connected with prolonged hospital stays, surgical morbidity (Tassoudis et al., 2011), and even death (Monk et al., 2015).

Previously, we proposed the iliopsoas space block technique to provide equivalent analgesic effects to the traditional posterior lumbosacral plexus approach (Dong et al., 2021), which was still paired with sacral plexus block of the supine posture. The lumbosacral trunk walks below the psoas major and above the sacroiliac joint (Miniato and Varacallo, 2022); therefore, bolus injection of regional anaesthetic into the iliopsoas space could theoretically block the lumbosacral trunk by spreading around the psoas major space. In this randomised clinical trial, it was hypothesised that the single iliopsoas muscle space block might provide older patients undergoing surgical repair for hip fractures with a decreased incidence of IOH and better analgesia comparable to traditional posterior lumbosacral plexus block.

## Materials and methods

### Study design

Between October 2021 and August 2022, we performed a double-blind, randomised, controlled trial to compare the hemodynamic effects of ultrasound-guided iliopsoas block and posterior lumbosacral plexus block in elderly patients with hip fractures undergoing surgery. The Shanghai Sixth People’s Hospital Ethics Committee (Ethical Committee Number 2021-221) approved the trial, which was registered at^1^ (ChiCTR2100051394, principal investigator: Tao Xu).

### Study population

Between October 2021 and August 2022, patients aged ≥65 years, with American Society of Anaesthesiologists (ASA) physical status class I-III and scheduled for unilateral hip surgery under general anaesthesia were recruited. All participants or their legal representatives gave their written, informed permission. Infection at the puncture site, history of hip surgery, preexisting neurological deficiency in the lower extremities, contraindications for regional anaesthesia, history of allergy to local anaesthetics, coagulopathy, substance addiction, or daily use of analgesics were exclusion criteria. Patients who refused to cooperate with the researchers were excluded from the study. Most surgeries for hip fractures took <2 h, while those that took longer were also excluded from the study.

### Randomisation

All individuals were randomly assigned to either the iliopsoas muscle space group or the posterior lumbosacral plexus group by a physician not blinded to the experimental condition. Every ultrasound-guided nerve block was administered by a professional anaesthesiologist. The next step was conducted by a research coordinator or investigator who was blinded while collecting the data.

### Anaesthetic management

All patients underwent fasting according to the ASA guidelines and were not premedicated. A peripheral intravenous access was established as well. The nerve block was administered by a senior anaesthesiologist with >10 y of expertise in ultrasound-guided nerve blocks. The skin of the block region was cleansed with iodophor and both the sterile window sheets and probe were covered with sterile drapes. A standardised amount of local anaesthetic was administered utilising ultrasound guidance and a portable ultrasound system (M-Turbo; SonoSite, Bothell, WA), a 2- to 5-MHz curved array transducer, and a 22-G needle (KDL; Shanghai KDL Medical Instruments, China). The impact and scope of the nerve blocks were assessed and documented. Using thermal imaging and infrared (IR) cameras (FLIR Systems, Wilsonville, OR), we measured bilateral lower limb skin temperature 20 min after completion of nerve block, based on previous research regarding the onset time of nerve block (Dong et al., 2021).

**Figure 1:**
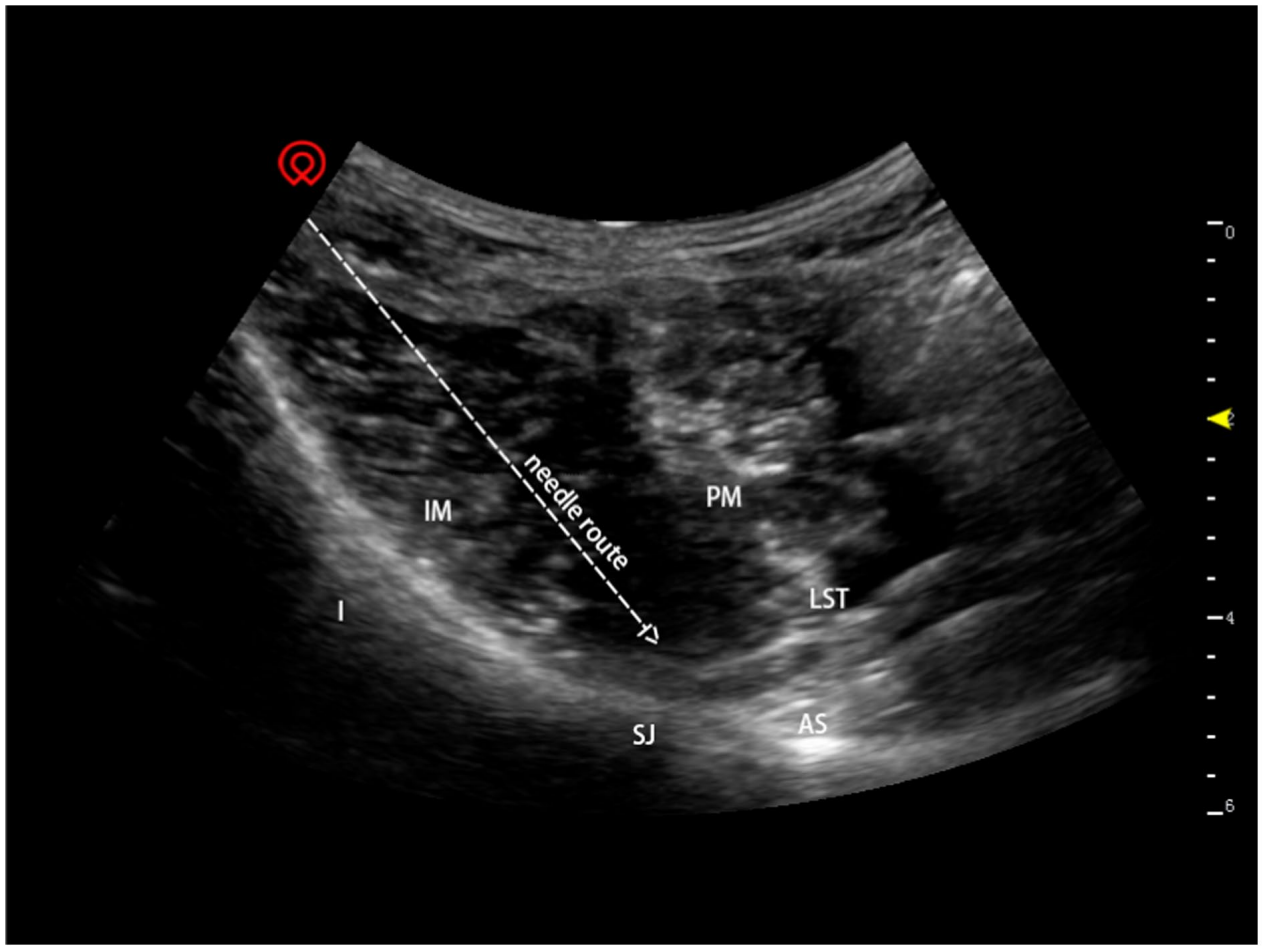
Ultrasound-guided anterior iliopsoas muscle space block. I: ilium; IM: iliac muscle; LST: lumbosacral trunk; PM: psoas muscle; SJ: sacroiliac joint; AS: alae sacralis.

In the posterior lumbosacral plexus group, the lumbar plexus block was conducted using 30 ml of 0.333% ropivacaine at L2-3 and L3-4 utilising a longitudinal paravertebral scan and out-of-plane injection approach, as described by Kirchmair et al. (2001). As documented by Bendtsen et al. (2014), the patients also underwent a sacral plexus block consisting 30 ml of 0.333% ropivacaine. In accordance with our previous procedure, a transverse-section ultrasound scan was used to guide the placement of 50 ml of 0. 333% ropivacaine in the lower region of the abdominal wall, 3–4 cm medial to the anterior superior iliac spine in a spine position (Figure 1; Dong et al., 2021) along with a lateral femoral cutaneous nerve block using 10 ml of 0.333% ropivacaine, as described by Thybo et al. (2016).

Propofol (2–3 mg/kg) and sufentanil (5 μg) were subsequently administered to establish general anaesthesia, and a laryngeal-mask airway (Auraonce; Ambu, Xiamen, China) was implanted. Sevoflurane administration and spontaneous breathing helped maintain the minimum alveolar concentration of sevoflurane between 0.8 and 1. Intraoperatively, a bolus dose of 2–3 μg of sufentanil was administered if the surgical stimuli caused an increase of 10 beats per minute (bpm) in heart rate or an increase of 20 mmHg in systolic blood pressure (SBP) at baseline. Sufentanil was adjusted to keep the respiratory rate < 20 bpm. The entire intraoperative sufentanil dosage was documented. During the perioperative phase, SBP, diastolic blood pressure (DBP), mean arterial pressure (MAP), heart rate (HR), and pulse oxygen saturation were recorded with invasive arterial blood-pressure monitoring.

The anaesthesiologist immediately delivers 3–5 mg ephedrine or 30-60 μg phenylephrine intravenously whenever IOH occurs, SBP <100 mmHg. The anaesthesiologist will administer 0.4–0.5 mg of atropine intravenously when the patient’s heart rate falls below 50 beats per minute. All cardiovascular drugs used during the surgery were documented.

Every patient was admitted to the post-anaesthesia care unit (PACU) after surgery. The nursing personnel and a study coordinator blinded to conditions assessed the pain score 60 min after surgery. Using a visual analogue scale (VAS) ranging from 0 to 10, pain ratings were assessed (0 = no pain, 10 = greatest agony). In the PACU, opioids were given intravenously as necessary.

### Outcomes

The definition of IOH remains controversial (Filiberto et al., 2021). Based on the Perioperative Quality Initiative (POQI) statement (Sessler et al., 2019) and related studies, we defined IOH as a SBP < 100 mmHg (Weinberg et al., 2022). As primary endpoints, a composite measure of IOH incidence, including frequency, absolute duration, and relative duration (percentage of total anaesthesia time), was established for this investigation, as stated by Schneck et al. (2020), while secondary endpoints included the pain score recorded 60 min after surgery and perioperative sufentanil dose.

### Sample size

Based on the clinical data from our studies, we deemed a difference (confidence interval) of 2 in the frequency of IOH to be statistically significant. Assuming a standard deviation (SD) of 1.75 in each group, 36 participants would give 90% power to deliver an iliopsoas space block, which is related with a lower incidence of IOH than a lumbosacral posterior plexus block for senior hip fractures. Considering a drop-out rate of 20%, 44 patients were required to be enrolled in this study.

### Statistical analysis

Demographic information, clinical features, and hemodynamic data were included in descriptive statistics. Non-normally distributed variables are shown as medians and interquartile ranges, as opposed to means and SDs for regularly distributed data. Numeral and percentage representations of categorical variables are used. Independent Student’s t-tests were used to analyse differences in main outcomes across groups for continuous variables, while non-parametric tests were used for nominal variables. The significance threshold was fixed at *p* < 0.05. GraphPad Prism 6 was used to conduct statistical analysis (GraphPad Software, San Diego, CA). Two one-sided test (TOST) protocols were used to assess the secondary outcome across groups and determine if they were equivalent (2 equivalency margin).

## Results

**Figure 2:**
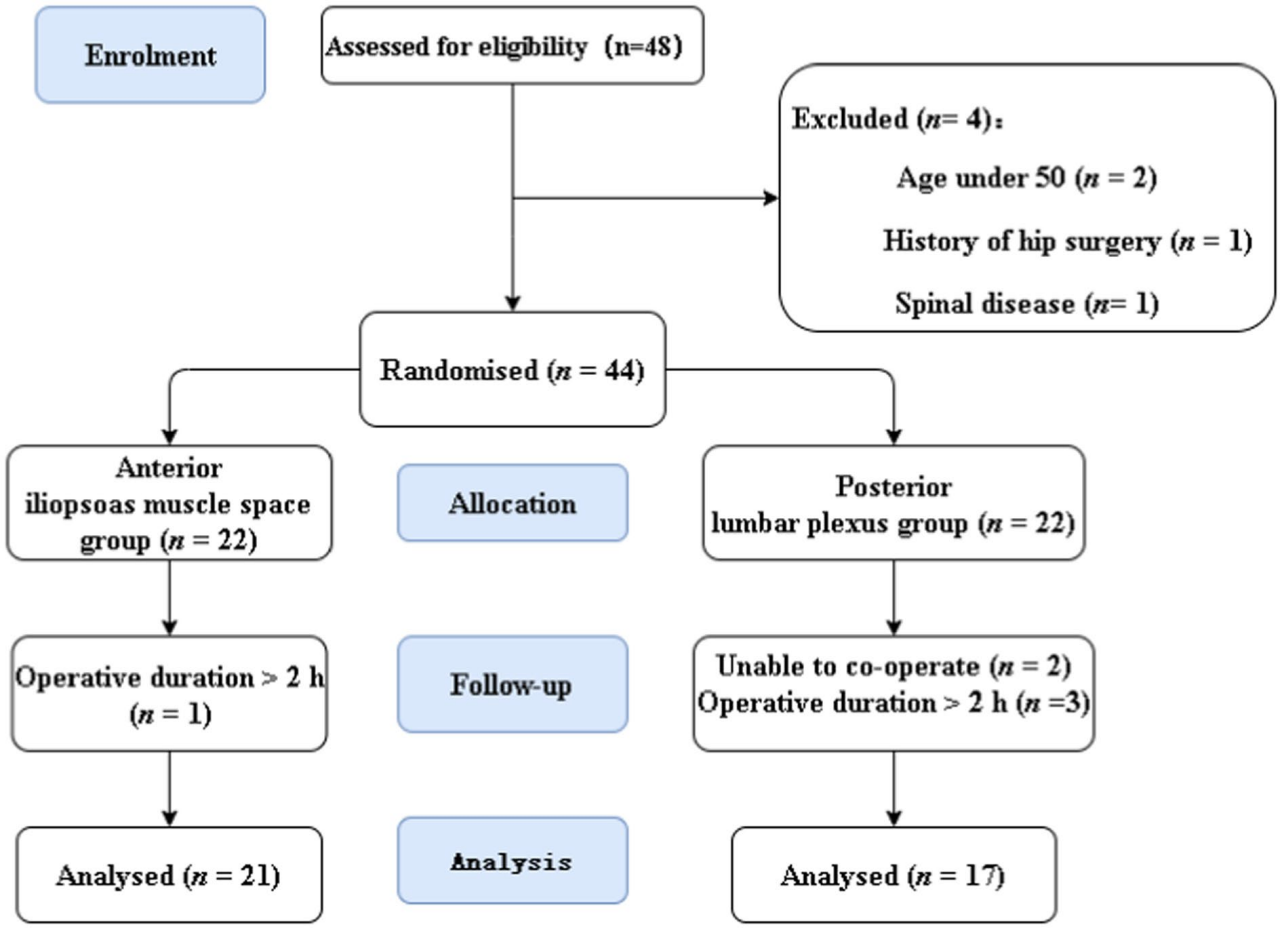
CONSORT flow diagram.

This research involved 48 individuals. Four individuals were excluded, and 44 patients were finally recruited in the trial (Figure 2). Six were excluded from analysis as the procedure lasted >2 h; 38 patients were included in the final analysis: 21 in the iliopsoas muscle space group and 17 in the posterior lumbosacral plexus group. Table 1 provides a summary of patient characteristics for the two groups.

**Table 1:** General patient and surgery characteristics.

Characteristics	Iliopsoas space group (*n* = 21)	Posterior lumbosacral plexus group (*n* = 17)	*p* value
Sex, F (M)	12 (9)	7 (10)	0. 328
Height (cm)	160. 00 ± 7. 16	164. 00 ± 7. 25	0. 097
Weight (kg)	58. 38 ± 10. 41	60. 71 ± 10. 05	0. 492
BMI (kg/m^2^)	22. 84 ± 3. 98	22. 47 ± 2. 63	0. 745
Age (years)	73. 33 ± 13. 10	76. 76 ± 9. 13	0. 367
Operation duration (min)	59. 05 ± 24. 18	62. 94 ± 18. 88	0. 591
Duration of general anaesthesia (min)	76. 57 ± 31. 46	79. 00 ± 20. 40	0. 785
PACU 1-h VAS score	2 (1–3)	1 (0–3)	0. 279
Perioperative propofol dose (mg)	130 (110–160)	130 (102–150)	0. 554
Perioperative dose of sufentanil (μg)	8 (6–10)	5 (5–8)	0. 176

Data are expressed as mean ± SD or median (interquartile range). SD: standard deviation; BMI: body mass index; VAS: visual analogue scale; PACU: post-anaesthesia care unit.

### Iliopsoas muscle space block reduces the incidence of IOH

**Figure 3:**
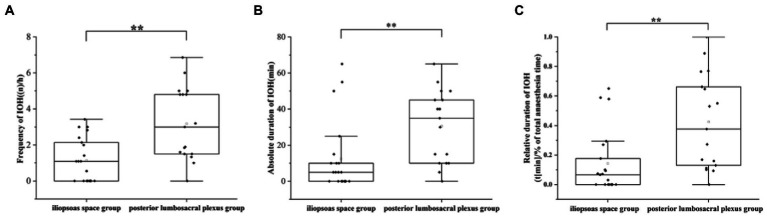
Graphs demonstrating the frequency **(A)**, absolute **(B)**, and relative duration **(C)** of hypotensive events in the study. IOH: intraoperative hypotension. **p < 0.01.

The IOH frequency was considerably lower in the iliopsoas space block group than that in the posterior lumbosacral plexus group {all values in median interquartile [IQR] and shown as numbers of hypotensive events per hour (n/h); 1.09 (0–2.14), 3 (1.6–4.8); *p* = 0. 001, respectively}. In comparison to the posterior lumbosacral plexus group, the iliopsoas space block group had considerably shorter absolute [5 (0–10) min, 35 (10–45) min; *p* = 0.008, respectively] and relative [minutes below SBP 100 mmHg in % of total anaesthesia time, 6.67 (0–17.65), 37.6 (12.99–66.18); *p* = 0.004, respectively] durations of IOH (Figure 3; Table 2). However, neither the number nor the duration of hypotension events (SBP < 90 mmHg) differed substantially between groups.

**Table 2:** Overview of primary endpoint parameters.

Characteristics	Iliopsoas space group (*n* = 21)	Posterior lumbosacral plexus group (*n* = 17)	*P* value
Frequency of IOH, n/h	1. 09 (0–2. 14)	3 (1. 6–4. 8)	0. 001**
Absolute duration of IOH (min)	5 (0–10)	35 (10–45)	0. 008**
Relative duration of IOH%	6. 67 (0–17. 65)	37. 6 (12. 99–66. 18)	0. 004**
Number of hypotension (SBP < 90 mmHg)	0 (0–1)	1 (0–2)	0. 238
Duration of hypotension (SBP < 90 mmHg) (min)	0 (0–5)	5 (0–15)	0. 181
Number of hypotension (SBP < 100 mmHg)	1 (0–2)	4 (2–4)	0. 001**
Duration of hypotension (SBP < 100 mmHg) (min)	5 (0–10)	35 (10–45)	0. 008**
Surgery type (no. of cases)			0. 264
Total hip arthroplasty	10	10	
Hemi-arthroplasty	7	3	
Internal fixation of intertrochanteric fracture of femur	0	2	
Internal fixation of intertrochanteric fracture of femur	2	0	
Internal fixation of femoral neck fracture	2	2	

Values are shown as median [interquartile range] or number. n/h: numbers of hypotensive events per hour; IOH: intraoperative hypotension; SBP: systolic blood pressure. **p < 0.01.

### Pain scores in the PACU

**Figure 4:**
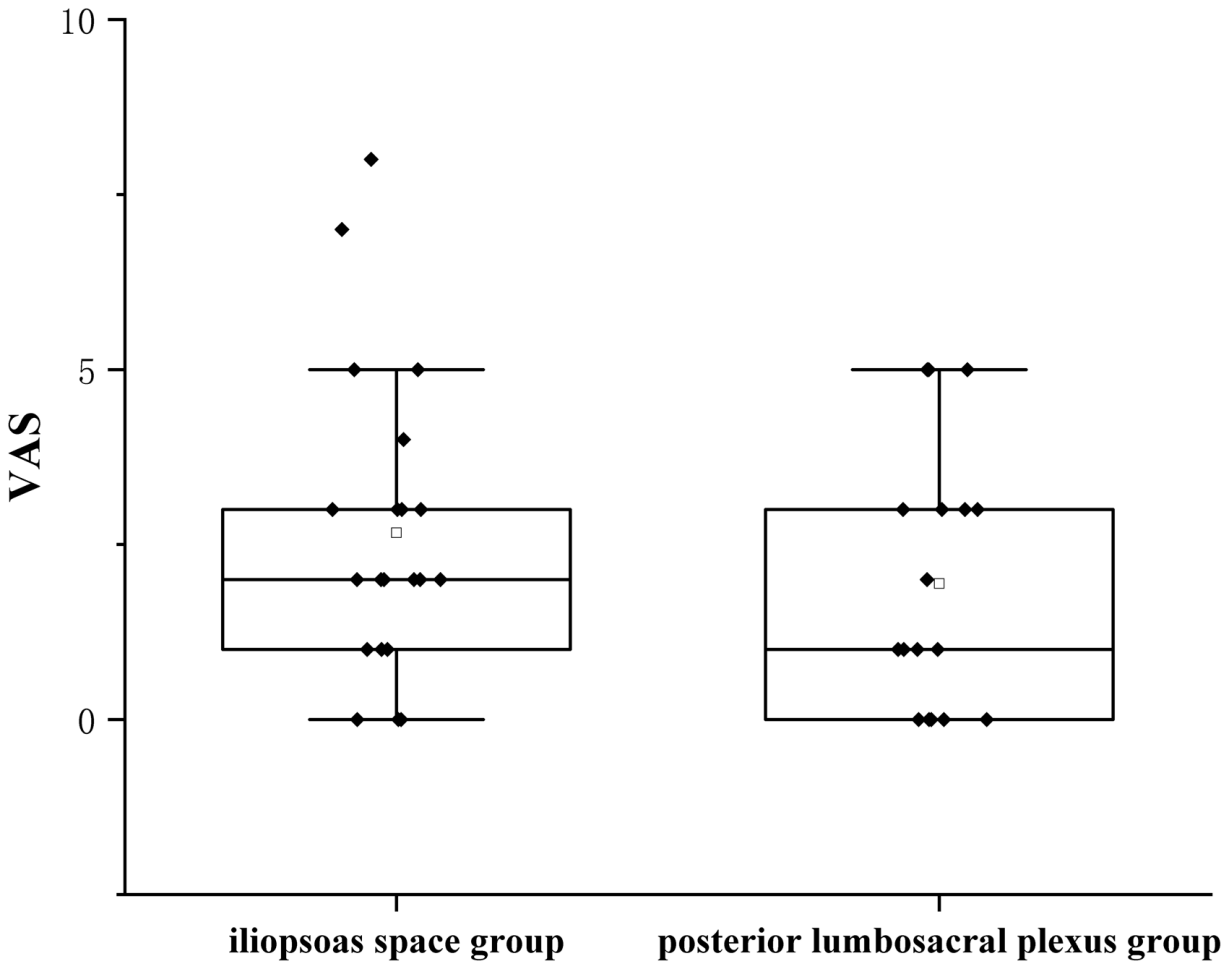
Effect of either an anterior iliopsoas space or posterior lumbosacral plexus block on postoperative pain after hip surgery with an injection of 0.33% ropivacaine. Data are expressed as median [interquartile range]. The 95% confidence intervals for the estimated group differences were prespecified tolerances and therefore deemed equivalent. VAS, visual analogue scale.

**Figure 5:**
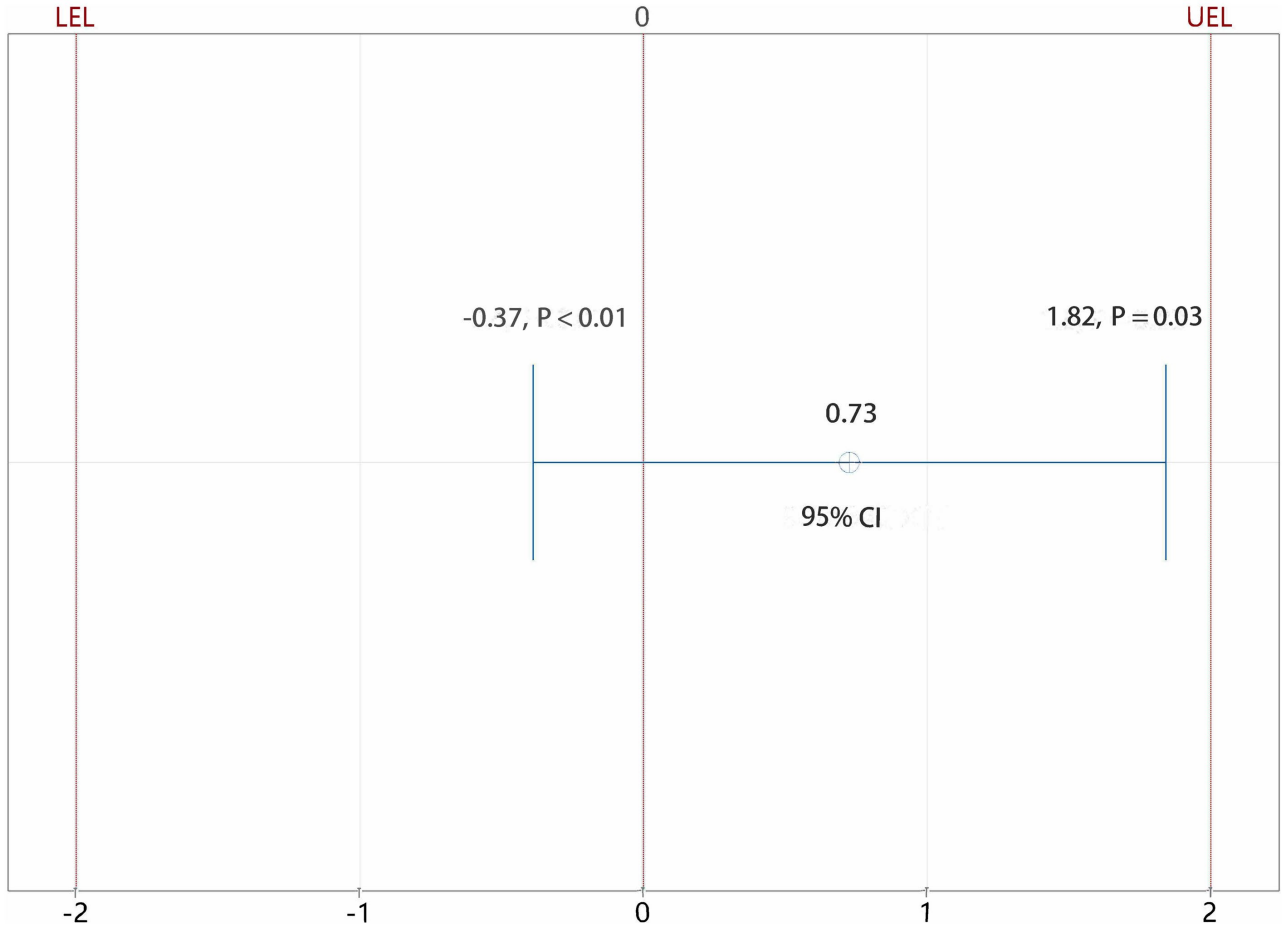
Equivalent test results of iliopsoas muscle space group and posterior lumbar plexus group. CI: confidence interval; LEL: lower equivalence limit; UEL: upper equivalence limit. Using the non-parametric TOST, the CI fell within the predetermined range of −2.0 to 2.0, the effects of the two therapies on postoperative pain were comparable.

Receivers of a posterior lumbar plexus block (*n* = 17) had a median IQR pain score of 1 (0–3), whereas those receiving an iliopsoas muscle space block (*n* = 21) received a score of 2(1–3), resulting in a group difference of −1 (95% confidence interval [CI], −0.614 to 2.065; *p* = 0.279; Figure 4). Since the CI fell within the predetermined range of-2.0 to 2.0, the effects of the two therapies on postoperative pain were comparable. Using the non-parametric TOST, equivalence was measured (Figure 5).

### Perioperative sufentanil usage

Sufentanil was administered in the iliopsoas space block group at a median IQR dose of 8 (6–10) μg and in the posterior group at a median IQR dose of 5 (5–8) μg (Table 1). There was no significant difference between the two groups (*p* = 0.176) indicating that both the iliopsoas space block and posterior lumbosacral plexus block may be useful for providing analgesia during hip surgery.

### Iliopsoas space block’s blocking range

FLIR imaging revealed the body surface temperature at the same selected points on the patients’ blocked and unblocked sides. The selected point temperature of the heel (sacral plexus innervation area) on the nerve-blocked side was 26.3°C while that of the unblocked side was 24.8°C. The temperature at the base of the foot (sacral plexus innervation area) on the blocked side was 25.7°C and that on the unblocked side was 23.4°C. The temperature at selected points of the distal femur (lumbar plexus innervation) on the nerve-blocked side was 27.2°C and that on the unblocked side was 26°C. The medial (lumbar plexus innervation region) side of the thigh had a temperature of 29.5°C whereas the unblocked side had 28.9°C. The darker the colour of the corresponding region, the lower the temperature. After the nerve block, the blocked sacral plexus innervation area (sciatic innervation area) had a brighter colour, indicating a higher skin temperature and nerve blockage, compared to the unblocked side (Supplementary Figure 1). After the nerve block was administered, the limb skin temperature of the region innervated by the femoral nerve, obturator nerve, and sciatic nerve was higher on the blocked side than on the unblocked side, indicating that both the lumbar plexus and lumbosacral trunk were successfully blocked.

## Discussion

Building on our previous work (Dong et al., 2021), we compared the incidence of hypotension and analgesic effects of a single iliopsoas space block to those of a posterior lumbosacral plexus block in hip surgery. Significantly lesser IOH occurred in the iliopsoas plexus group than in the posterior lumbosacral plexus group. In addition, the pain relief after surgery was comparable across groups, indicating that a single iliopsoas space block not only minimises the incidence of IOH but also has the same pain-relieving effect as a posterior lumbosacral plexus block.

Posterior lumbosacral plexus block is often utilised in hip surgery; however, IOH frequently occurs and has unanticipated outcomes (Aksoy et al., 2014; Zhang et al., 2022). Evidence has demonstrated that longer periods of SBP < 100 mmHg following non-cardiac surgery are related with an increased risk of organ damage and death (Sessler et al., 2019). Based on Weinberg et al. (2022), 153 studies (48.1%) did not include any duration variable in the definition of hypotension; however, IOH should be defined using the absolute values indicated in the POQI statement (Sessler et al., 2019); that is, MAP < 60–70 mmHg or SBP < 100 mmHg. Accordingly, we defined IOH as SBP < 100 mmHg in the current study. Here, we demonstrated that the incidence of IOH considerably decreased in elderly patients undergoing hip fracture surgery using an iliopsoas space block compared to the posterior lumbosacral plexus block.

Possible causes of IOH after traditional posterior lumbosacral plexus block include nerve root block, local anaesthetics’ epidural space diffusion, sympathetic nerve block, and excessive analgesia (Lee and Braehler, 2017). When performing a lumbosacral plexus block, if the needle point is placed extremely near the foraminal opening, the medication may transfer down the foraminal to the epidural area, resulting in an epidural block (Lee and Braehler, 2017). Furthermore, since the lumbar sympathetic nerve is positioned between the anterolateral lumbar vertebrae and the medial psoas major muscle (Feigl et al., 2011), it may be blocked simultaneously during the lumbar plexus block. Above all, a lumbosacral plexus block may cause blood vessel dilation of the lower extremities and blood pressure decline, which happens in up to 81% of non-cardiac procedures and is related with a higher 30-day death rate (van Waes et al., 2016). A single anterior iliopsoas space block can effectively avoid these potential problems. With this newly introduced technique, the local anaesthetic rarely enters the epidural diffusion, and the block is relatively restricted. Compared with the posterior lumbosacral plexus, the anterior iliopsoas space block migrates more to the periphery to achieve accurate block and provide relative analgesia without being excessive. Therefore, an iliopsoas anterior space block can effectively decrease the occurrence of IOH and provide an appropriate analgesic effect to the elderly during hip surgery.

As the lumbosacral trunk passes medially to the psoas major muscle at the level of the first sacral vertebra (Miniato and Varacallo, 2022), it can be blocked by injecting a high-volume of local anaesthetic into the iliopsoas space, diffusing medially into the psoas major. Despite the fact that the iliopsoas space block requires a longer onset time than the posterior lumbosacral plexus block (Dong et al., 2021), the iliopsoas space block can not only block the femoral nerve, obturator nerve, and lateral femoral cutaneous nerve, but also the lumbosacral trunk, thereby providing adequate analgesia for hip fracture surgery without additional sacral plexus block. However, variations in the abnormally high VAS score 1 h after surgery also indicate that the possible local anaesthetic diffusion in the space around the psoas major muscle may also result in imperfect blockade.

In addition, the anterior iliopsoas block has many other advantages over the posterior lumbosacral plexus block, such as avoiding the risk of cardiovascular disturbances caused by the pain of turning over in the fracture patient, risk of thrombus dislodging from the leg vein caused by turning over, and risk of aggravated fracture injury caused by postural changes. Additionally, the anterior iliopsoas block may minimise the number of punctures and/or repeated blocks (sacral plexus), and the occurrence of blockage-related haemorrhage, nerve damage, and total spinal anaesthesia. The anterior block provides separation from the abdominal cavity to protect the kidneys and intestines from possible injury. Therefore, it is beneficial to promote anterior iliopsoas space block in the supine posture.

The present research has certain limitations. First, propofol causes hypotension following anaesthesia induction; on the other hand, age-related changes in vascular tone, homeostatic disturbances, and sympathetic nervous system tonic activity render the elderly more vulnerable to IOH. The perioperative bispectral index should be monitored to avoid the effect of anaesthesia depth on blood pressure fluctuations. Second, local anaesthetic concentration and volumetric titration were not assessed to obtain more accurate injection volumes and concentrations.

In conclusion, this study demonstrated that a single iliopsoas muscle space block not only provides as much perioperative analgesia as the traditional lumbosacral plexus block, but also reduces the number of punctures, helps avoid position changes of the patient, and effectively reduces the incidence of IOH, thus improving patient prognosis and increasing perioperative safety.

## Data availability statement

The original contributions presented in the study are included in the article/Supplementary material, further inquiries can be directed to the corresponding author.

## Ethics statement

The Shanghai Sixth People’s Hospital Ethics Committee (Ethical Committee Number 2021-221) approved the trial. The patients/participants provided their written informed consent to participate in this study.

## Author contributions

TX conceived and designed the experiments. The data collection was performed by QT, HY and JD. QT and CW analysed the data. The manuscript was written by TX, QT and CW. MC contributed materials and analysis tool. All authors contributed to the article and approved the submitted version.

## Funding

This work was supported by grant from the Natural Science Foundation of China (82171486), the Interdisciplinary Program of Shanghai Jiao Tong University to TX (YG2021ZD23), General Science Foundation of Shanghai Sixth People’s Hospital to TX (YNMS202114) and Scientific research fund of Shanghai Sixth People’s Hospital to MC (YNTS202005).

## Conflict of interest

The authors declare that the research was conducted in the absence of any commercial or financial relationships that could be construed as a potential conflict of interest.

## Publisher’s note

All claims expressed in this article are solely those of the authors and do not necessarily represent those of their affiliated organizations, or those of the publisher, the editors and the reviewers. Any product that may be evaluated in this article, or claim that may be made by its manufacturer, is not guaranteed or endorsed by the publisher.

## Footnotes

^1^ http://www.Chictr.org.cn
