# Supplementary material for: Ultrasound-guided anterior iliopsoas muscle space block effectively reduces intraoperative hypotension in elderly adults undergoing hip surgery: A randomised controlled trial
Source: Front Mol Neurosci. 2023 Jan 23;16:1119667. doi: 10.3389/fnmol.2023.1119667 (PMC9900129; doi:10.3389/fnmol.2023.1119667)
Supplement: Supplementary file 1 [file Data_Sheet_1.docx]

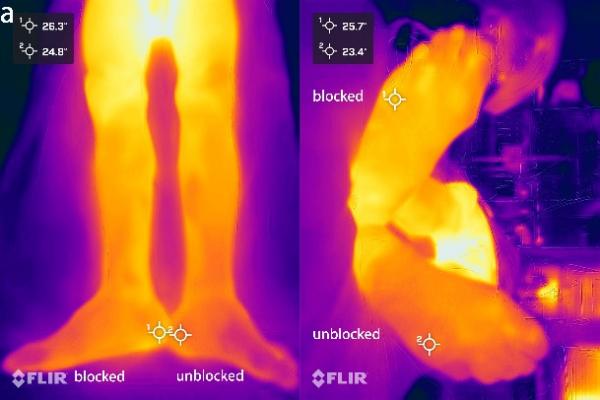

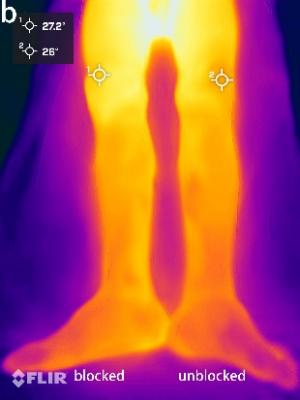

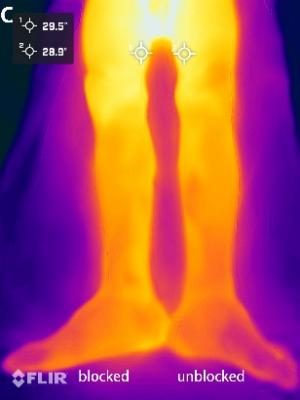


Supplemental Fig. 1 Lower extremity thermography after anterior iliopsoas muscle space block, showing the temperature of selected points at the same location in the same innervation area on both lower limbs. (a) Area innervated by the sciatic nerve. (b) Area innervated by the femoral nerve. (c) Area innervated by the obturator nerve.
